# Identification and Molecular Characterisation of a Novel Mu-Like Bacteriophage, SfMu, of *Shigella flexneri*

**DOI:** 10.1371/journal.pone.0124053

**Published:** 2015-04-22

**Authors:** Richa Jakhetia, Naresh K. Verma

**Affiliations:** Division of Biomedical Science and Biochemistry, Research School of Biology, The Australian National University, Canberra, ACT 0200, Australia; ContraFect Corporation, UNITED STATES

## Abstract

*S*. *flexneri* is the leading cause of bacillary dysentery in the developing countries. Several temperate phages originating from this host have been characterised. However, all *S*. *flexneri* phages known to date are lambdoid phages, which have the ability to confer the O-antigen modification of their host. In this study, we report the isolation and characterisation of a novel Mu-like phage from a serotype 4a strain of *S*. *flexneri*. The genome of phage SfMu is composed of 37,146 bp and is predicted to contain 55 open reading frames (*orfs*). Comparative genome analysis of phage SfMu with Mu and other Mu-like phages revealed that SfMu is closely related to phage Mu, sharing >90% identity with majority of its proteins. Moreover, investigation of phage SfMu receptor on the surface of the host cell revealed that the O-antigen of the host serves as the receptor for the adsorption of phage SfMu. This study also demonstrates pervasiveness of SfMu phage in *S*. *flexneri*, by identifying complete SfMu prophage strains of serotype X and Y, and remnants of SfMu in strains belonging to 4 other serotypes, thereby indicating that transposable phages in *S*. *flexneri* are not uncommon. The findings of this study contribute an advance in our current knowledge of *S*. *flexneri* phages and will also play a key role in understanding the evolution of *S*. *flexneri*.

## Introduction

A large number of mobile DNA elements play an important role in the evolution of bacterial genomes. These elements can translocate from site to site within and between bacterial genomes, generating insertional mutations and various types of genome rearrangements, thereby creating an important source of genetic variation. These transposing elements include IS element, composite transposons and some bacteriophages which propagate via transposition [1,2].

Enterobacteria phage Mu of *Escherichia coli* is the best studied transposable phage. It is known to randomly integrate into the host chromosome during both lytic and lysogenic developments often leading to mutations in the host [3,4]. In addition, phage Mu carries heterogeneous host DNA sequences on the ends of the packaged genome which results in transduction of variable amounts of host DNA [5]. These properties make bacteriophage Mu an important tool for genetic research.

With the exception of *Pseudomonas*, for which over 60 transposable phages have been reported [6], only a few functional Mu-like phages have been isolated from other bacteria. These are *E*. *coli* phage D108 (closely related to phage Mu) [7], *Burkholderia cenocepacia* phage BcepMu and KSI0 [8,9], the recently isolated *Rhodobacter capsulatus* phage RcapMu [10], and *Haemophilus parasuis* phage SuMu [11]. However, Mu-like prophage elements have been identified in several other Gram-negative bacterial genomes such as *Haemophilus influenzae* (FluMu), *Neisseria meningitidis* (Pnm1), *Deinococcus radiodurans* R1 (RadMu) [12], *Shewanella oneidensis* (MuSo1 and MuSo2) [13], and *E*. *coli* O157 Satai (Sp18) [14]. Although phages within each bacterial genus appear to share homology, only a limited proteome correlation has been found between phage Mu and Mu-like phages from a different bacterial genus.

Genus *Shigella* belongs to the family *Enterobacteriaceae*, and is the causative agent of shigellosis or bacterial dysentery. It is responsible for more than 165 million cases annually, of which 1.1 million results in death. Among the four known species of *Shigella*, *S*. *flexneri* is the primary cause of endemic shigellosis prevalent in the developing countries, and is the most frequently isolated species world-wide [15]. Based on the structure of the lipopolysaccharide O-antigen, *S*. *flexneri* is divided into 19 serotypes [16]. To date, seven temperate bacteriophages originating from various serotypes of *S*. *flexneri* have been isolated. All of these carry the O-antigen modification genes and mediate serotype conversion by integration into the host chromosome. Moreover, based on their genome organization, they are known to be members of the lambdoid family of phages [17–22].

In this study, we report the isolation and characterisation of a novel Mu-like bacteriophage, SfMu, from a wild type serotype 4a strain of *S*. *flexneri*. Comparative genome analysis revealed high level of proteome correlation between bacteriophage SfMu and Mu. Analysis of SfMu cell wall receptor indicates that the phage SfMu uses O-antigen of the LPS as its receptor. Additionally, this study also identified cryptic and complete SfMu prophage in various serotypes of *S*. *flexneri*, suggesting that Mu-like phages are not uncommon in *S*. *flexneri*.

## Materials and Methods

### Bacterial strains, bacteriophage and media

Bacteriophage SfMu was induced from the serotype 4a strain of *S*. *flexneri*, SFL2241, using UV irradiation protocol described by Adam et al [23]. Bacteriophage stocks were prepared by propagating the induced lysate on serotype Y strain (SFL124) [24], and precipitating phage using the polyethylene glycol, as described in Sambrook et al [25].

Luria-Bertani (LB) broth or LB agar was used for culturing of bacteria and NZCYM was employed for propagation of the phage.

### Electron microscopy

Phage particles were allowed to absorb onto the carbon-coated copper grid before they were negatively stained with 2% phosphotungstenic acid (pH 7.0) and examined under a Hitachi H7000 transmission electron microscope.

### Plaque assay modified with Antibiotic (PAMA)

Phage SfMu plaque assays were performed using the PAMA technique described by Santos et al [26]. Briefly, 0.1 ml of phage lysate was mixed with 0.1 ml of stationary phase culture of the host bacterium, and incubated at 37°C for 20 min. The suspension was then mixed with 3 ml of LB top agar containing 10% glycerol and 0.5 mg/l ampicillin, and poured onto a LB agar plate supplemented with 10% glycerol and 0.5 mg/l ampicillin. Plates were allowed to dry and then incubated overnight at 37°C.

### DNA techniques

Bacterial genomic DNA was isolated using GE Healthcare genomic DNA isolation kit (GE Healthcare), according to the manufacturer’s instructions. Restriction enzymes were obtained from New England Biolabs (NEB) and used according to the manufacturer’s directions. PCR amplification was performed using the PfuUltra II Fusion HS DNA Polymerase (Stratagene). When necessary, the PCR products were purified by using the Wizard SV Gel and PCR Clean Up System (Promega). Sequencing of the purified products was performed using Big Dye Terminator v3.1 Cycle Sequencing Kit and were run at the Biomedical Resources Facility, John Curtin School of Medical Research, Australian National University.

Multiplex PCR amplifications were performed in 20 μl volumes with 0.125 μM of each primer, 2 Units Taq DNA polymerase, 2.25 mM MgCl_2_, 1 X Taq buffer, 50 ng DNA, and 200 μM of dNTP mix. DNA amplifications were performed under the following conditions: initial denaturation at 95°C for 2 min, followed by 35 cycles of denaturation at 95°C for 30 s, annealing at 55°C for 30 s, and extension at 72°C for 3 min. The final extension was carried out at 72°C for 5 min. PCR products were analysed by electrophoresis using 0.7% agarose gels.

For Southern hybridization, 1 μg of genomic DNA was digested with *Eco*RV restriction enzyme and subjected to electrophoresis on a 0.7% agarose gel. Capillary blotting was then used to transfer DNA onto a positively charged nylon membrane [25]. The membrane was hybridized using *Eco*RV digested SfMu phage DNA probe prepared using PCR DIG labeling mix (Roche). Pre-hybridization, hybridization, washes and detection (CDP-star) were performed as recommended by Roche.

### Phage inactivation

LPS was isolated from dried bacterial cells using the phenol water extraction method [27]. Phage inactivation assay was performed using the protocol described by Sandulache et al [28] with the following modifications. 0.1 ml of LPS (5 to 100 μg) was mixed with 0.1 ml of phage lysate (1x10^4^ PFU/ml in LB) and incubated at 37°C for 1 hr. 0.2 ml of stationary phase culture of the indicator strain (SFL124) was then added to the mixture. Following a further incubation for 20 min at 37°C, the mixture was plated using the PAMA technique described above. Plaques were counted after incubating the plates overnight at 37°C.

### Phage genome sequencing, annotation and analysis

Complete sequence of phage SfMu was determined by sequencing the genome of its host strain SFL2241 using 250 bp paired end, Miseq, Illumina sequencing, at Ramaciotti Centre, University of New South Wales. The reads generated were de-novo assembled into contigs using Velvet [29]. The gap between the contigs containing phage SfMu genome was closed by amplifying the desired region and sequencing the purified PCR product as described above.

The open reading frames were identified using CLC Main Workbench (Ver 5.5.1, CLC Bio) and NCBI ORF finder program. All predicted *orfs* were corroborated by the inspection of the Shine-Dalgarno sequence and by homology searches against GenBank using the BlastP algorithm. The tRNAscan-SE program was used to search for tRNA genes [30], and the Rho-independent terminators were identified using ARLOND terminator finding program [31].

Whole genome alignments were conducted with Mauve [32], and the protein level alignments were performed using ClustalW [33]. The accession numbers for the phages used for comparative genomics were: Mu *cts*62 (NC_000929), D108 (NC_013594), B3 (NC_006548), BcepMu (NC_005882), KS10 (NC_011216), RcapMu (NC_016165), SuMu (NC_019455).

### Accession Number

The nucleotide sequence of SfMu phage reported in this article has been deposited in the GenBank database as accession number KP010268.

## Results and Discussion

### Isolation of SfMu

**Fig 1:**
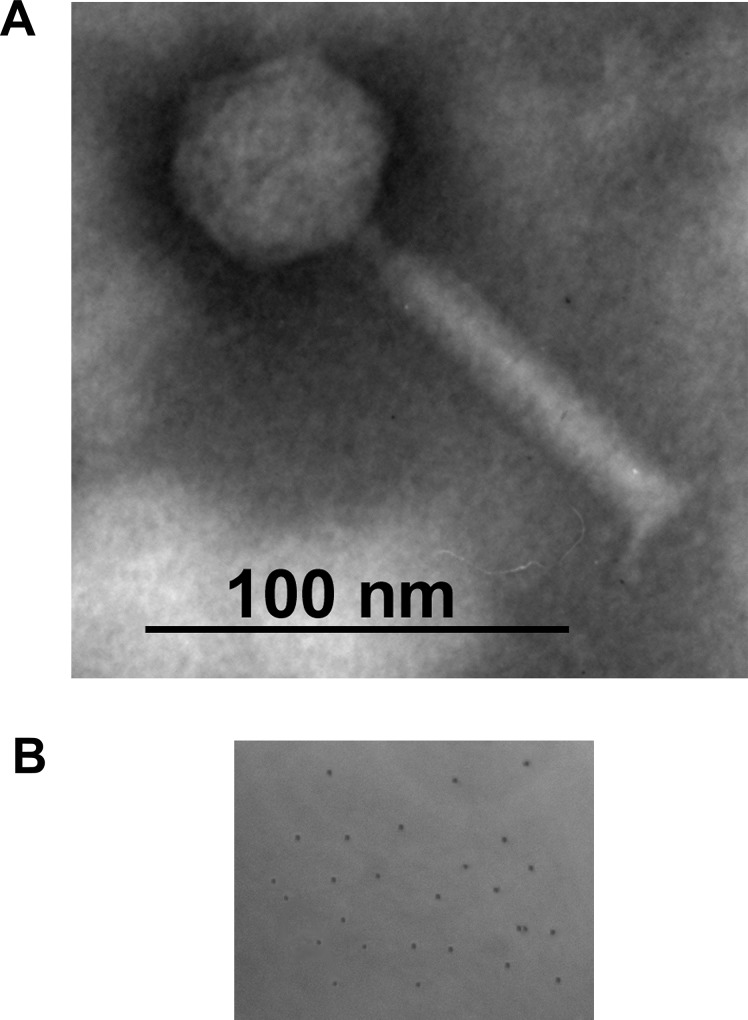
Morphology of bacteriophage SfMu. A) Electron micrograph of phage SfMu, negatively stained with 2% phosphotungstenic acid. Scale bar 100 nm. B) Plaques of phage SfMu.

Bacteriophage SfMu was isolated from the serotype 4a strain, SFL2241. Cell lysis was observed after induction (phage yield: 1x10^4^–10^5^ PFU/ml) and on propagating the induced lysate on SFL124 (serotype Y, indicator strain). However, phage SfMu plaques were only visible when the lysates were plated onto the indicator strain using the PAMA technique [26]. The plaques obtained had a clear pin point morphology with well-defined boundaries (Fig 1).

In order to confirm that SfMu is a prophage of SFL2241, Southern hybridization experiments using DIG-labelled SfMu as a probe were performed on *Eco*RV digested genomic DNA of SFL2241. Results confirmed the presence of phage genome integrated into the host chromosome (S1 Fig).

### Morphology

Electron micrographs (EM) of negatively stained SfMu virions reveal an icosahedral head and a contractile tail (Fig 1). These characteristic features are typical of group ‘A’ phages of family *Myoviridae* and order *Caudovirale*, according to the morphological classification of Bradley [34]. The average particle had a head size of approx. 50 nm and a tail length of approx. 100 nm, which is comparable to that of bacteriophage Mu (head size 54 nm and tail 100 nm).

### Genome features of bacteriophage SfMu

The complete genome sequence of bacteriophage SfMu was determined by sequencing the DNA of its host (SFL2241), using Illumina (Miseq). The data generated contained approx. 4,733,416 reads with avg read length of 250 bases, which were de-novo assembled into 1500 contigs, using Velvet. The accuracy of contigs was verified by mapping the reads back to the contigs. SfMu phage genome was found to be distributed between 2 contigs. The gap between the two contigs was then closed by targeted PCR and subsequent sequencing to yield a single contiguous sequence of phage SfMu genome totalling 37,146 bp in length. Phage SfMu genome has a G+C content of 51.9% which is comparable to its host (approx. 51%).

**Fig 2:**
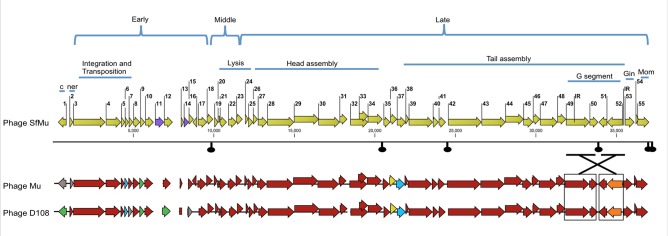
Genome of bacteriophage SfMu. The genome of phage SfMu is shown with a scale in bp. Predicted *orfs* are designated with arrows, pointing in the direction of transcription. Putative functional modules are indicated above the arrows. Black knobs indicate the position of predicted terminators. Comparison of SfMu encoded proteins to their counterparts in phage Mu and D108 is shown below the scale. Different colours show the level of amino acid identity: red: 90–100% identity, yellow: 80–89% identity, orange: 70–79% identity, green: 60–69% identity, blue: 50–59% identity and grey: 10–50% identity. The purple arrows in the SfMu map represent SfMu proteins which have no homologues in phage Mu or D108.

Analysis of phage SfMu genome revealed 55 putative open reading frames with a plausible Shine-Dalgarno sequence (Fig 2). Out of 55, only 4 transcribed leftwards and 51 transcribed rightwards. In addition, 49 of the predicted *orfs* initiated from ATG start, while 6 others used a GTG start. Phage SfMu genome is densely packed with the coding sequences occupying 94.2% of the genome. Several overlapping genes are present in phage SfMu genome, but minimal overlap of the start and stop codons was observed. The maximum overlap of 62 bp was detected between *orfs15-16*.

Proteins encoded by the predicted *orfs* were scanned for homologues using BlastP. Based on the similarities, possible functions were assigned to 29 ORFs and the other 26 ORFs showed similarity to uncharacterized proteins (S1 Table). Phage SfMu genome was also analysed for regulatory sequences and was found to contain six putative rho-independent transcription terminators (Fig 2). However, no tRNA genes were identified.

### Comparison of bacteriophage SfMu with phage Mu and D108

BlastP analysis of phage SfMu proteins also revealed that this phage contains genes encoding: transcriptional regulator (Ner), transposase, G segment invertase (Gin), etc, which are well known features of bacteriophage Mu, suggesting that SfMu is a Mu-like phage. DNA level comparison of phage SfMu was thus made with bacteriophage Mu and 6 other Mu-like phages from various hosts (*E*. *coli*, *P*. *aeruginosa*, *B*. *cenocepacia*, *R*. *capsulatus*, and *H*. *parasuis)*, using Mauve. The results of multiple alignment revealed that the genome of phage SfMu was highly similar to that of bacteriophage Mu and its closest relative phage D108, with conserved genomic and synteny characteristics. However, similarity of phage SfMu to the other Mu-like phages was of limited extent (S2 Fig).

To get an insight into the extent of similarity between phage SfMu and phage Mu or D108, SfMu encoded proteins were compared to their counterparts in phage Mu and D108. However, as phage D108 has been studied in less detail, most of the discussion in this report is based on phage Mu homologues. As shown in Fig 2, out of 55, 46 and 44 proteins of phage SfMu were >90% identical to their cognates in phage Mu and D108, respectively. Additionally, the arrangement of genes in phage SfMu was similar to bacteriophage Mu, and was divided into functional modules present in the following order from the left end: immunity, integration and transposition, lysis, head assembly and tail assembly. Moreover, based on the level of transcription during phage Mu’s lytic cycle, phage SfMu genome can also be subdivided into early, middle and late regions.

The left end of the phage SfMu genome encodes the regulatory proteins, C-repressor and Ner, which are involved in the regulation of the lytic and lysogenic developments of the phage. While phage SfMu C-repressor and Ner proteins shared 89% and 98% identity to their counterparts in phage D108, identity of only 24% and 47% was obtained with the phage Mu equivalents (Fig 2). Since the C-repressor in phage Mu is required for the establishment and maintenance of lysogeny [35], the difference in the repressor proteins of phage Mu and SfMu agrees with the fact that unlike phage Mu lysogens which can only be induced by heat [36], SfMu lysogens were induced by UV. Two other proteins in the early region were found to be different to their cognates in phage Mu. These were ORF6 and ORF9, proteins of unknown function, which shared 77% and 87% identity, respectively, with their equivalents in both phages Mu and D108. Additionally, ORF7 and 15 of phage SfMu showed <50% identity to their equivalents in D108, however, were identical to the phage Mu homologues.

ORF11 and 14 of phage SfMu are two proteins which have no homologues in phages Mu or D108, thus making these proteins unique to phage SfMu. However, bacteriophages Mu and D108 also contain two proteins (encoded by *orfs 12* and *19* in phage Mu, and *orfs 12* and *18* in phage D108) which do not share homology with any of the proteins in phage SfMu. As the two unique proteins of phages SfMu and Mu or D108 belong to the same region of the phage genome, they may be divergent homologues having conserved function. However, it is equally possible that they have different functions and each provides some benefit to the host.

The majority of the structural genes in the late region appear to be conserved between the three phages. The only exceptions are ORFs36-37 of phage SfMu, which share 50% and 70% identity, respectively, with their counterparts in both phages Mu and D108 (Fig 2). *orf36-37* are located between the head-gene module and the tail-gene module. Studies in phage Mu have revealed that the mutants defective in *orf36* produced full heads, unattached tails, and served as tail donors in *in vitro* complementation assays [37,38], thus suggesting that ORF36 protein might be involved in maturation of heads to allow joining of the tails. In addition, the mutants defective in *orf37* were shown to produce abnormally long tails and served as head donors in the complementation assay, suggesting ORF37’s involvement in tail formation or stabilization [37,38]. Although ORF36-37 of phage SfMu were different to their phage Mu counterparts, results of BlastP analysis revealed that homologues of these two proteins were present on a cryptic Mu-like prophage in *S*. *boydii*.

Proteins encoded by *orf49-52* of phage SfMu show homology to phage Mu G region. Genes *S*, *U*, *U’* and *S’* span the G segment in phage Mu. The proteins encoded by these genes are responsible for the tail fiber biosynthesis and assembly, and confer the host-range specificity to the phage [39,40]. Moreover, this segment lies next to the *gin* gene which encodes for a protein that promotes inversion of the G segment and therefore determines which pair of the tail fiber biosynthesis and assembly genes is expressed: *S* and *U* or *S’* and *U’* [41]. Based on the orientation of the G region, two types of phage particles are produced, Mu G(+) and Mu G(-), which differ in their host range. Phage with the G in (+) orientation (expressing *S* and *U* genes) are capable of infecting *E*. *coli* K12 [28], while those with the G(-) orientation (expressing *U’* and *S’* genes) infect *E*. *coli C*, *Enterobacter cloacae*, *Serratia marcescens*, *Citrobacter freundii*, *Erwinia* and *Shigella sonnei* [42]. The ‘G-segment’ of SfMu phage matches with that of phage Mu in G(-) orientation i.e. SfMu ORF49, 50, 51, and 52 show 96%, 97%, 90% and 65% identity to phage Mu S’, U’, U, and S proteins, respectively.

### Host Range of bacteriophage SfMu

In order to examine the sensitivity of phage SfMu, strains of *E*. *coli* K-12 and 12 serotypes of *S*. *flexneri* were tested by the PAMA method. Using the SfMu lysate produced after induction, phage SfMu was able to infect only serotypes Y and 3b strains of *S*. *flexneri*. No plaques were observed on *E*. *coli* K-12 strain or other serotypes of *S*. *flexneri*. The sensitivity to various serotypes of *S*. *flexneri* was also determined using SfMu lysate prepared upon infection on serotype Y strain. Interestingly, the host range of phage SfMu remained the same under both conditions. In bacteriophage Mu, lysates obtained upon induction are known to contain both types of phage particles, Mu G(+) and Mu G(-), in equal ratios. Moreover, in the lysates grown by infection, the majority of the phage particles have ‘G’ in one orientation [36]. As phage SfMu genome contains phage Mu like G segment, it was expected that the lysate obtained upon induction will have two kinds of phage particles (with G in either orientation) infecting different host, thus will show a broader host range than the lysate produced upon induction (containing one kind of phage particles).

The reason for the same host range observed with either lysates of phage SfMu could be because of no inversion in the G segment of phage SfMu. Like phage Mu, SfMu genome contains 34 bp inverted repeats flanking the G segment and is the target for Gin-mediated site specific recombination. Analysis of phage SfMu IR repeats revealed a 1 bp mutation on one of the IR sequences. Since this base change is present in only one of the sequences and it lies next to the two central bases which serve as a possible crossover site in the IR of phage Mu [43,44], it could result in the reduced rate or abolishment of the G segment inversion. However, it is also possible that the same host range is observed because phage SfMu particles in the G(-) orientation do not infect *S*. *flexneri* strains and are infectious for different species of bacteria which were not used in our analysis.

As mentioned earlier, the tail fiber proteins of phage Mu determine its host range specificity. Since ORF49 of phage SfMu is >95% identical to phage Mu S’ protein, we tested if phage Mu G(-) was able to infect *S*. *flexneri* like phage SfMu. Bacteriophage Mu was induced from its *E*. *coli* lysogen and the lysate obtained was tested for sensitivity against the 12 serotypes of *S*. *flexneri*. However, no plaques were obtained on any of the serotypes. Since phage Mu induced lysate contains both Mu G(+) and G(-) particles [36], it can be concluded that neither Mu G(+) nor G(-) infects *S*. *flexneri*. This suggests that although phage SfMu ORF49 and Mu S’ share a high sequence similarity, the structure of the two tail fibers is diverse enough to confer host specificity. The results of pairwise alignment of SfMu ORF49 with the S’ protein of bacteriophage Mu revealed 18 amino acid changes in ORF49 of SfMu phage (S3 Fig). Moreover, the first 147 amino acids of the two proteins were conserved, and most of the changes identified were in the carboxy terminal part of the protein. Out of 18, 7 amino acids were replaced by amino acids with similar properties, 5 polar and 2 nonpolar amino acids were replaced by a charged amino acid, 1 negatively charged amino acid was replaced by a nonpolar amino acid, while 2 nonpolar and 1 negatively charged amino acids were replaced by polar amino acids. The conserved N-terminal portion of the two tail fibers suggests that this part of the protein interacts with the phage tail, while the variable C-terminal is responsible for host recognition. This is similar to other *Myoviridae* phages like T4-like phages [45,46], *Lactococcus lactis* phages (sk1, TP901-1, and bIL170) [47], *Pseudomonas* phages (Pap1 and JG004) [48], and bacteriophage lambda [49], where the C-terminal domain of the tail fiber protein is responsible for binding to the host receptor.

### Cell wall receptor of bacteriophage SfMu

Since bacteriophage Mu uses distal part of the LPS core as its receptor [28,42], we sought to investigate if the primary receptor of SfMu is also located in the LPS. Plaque assays using the PAMA technique were performed using a mutant serotype Y strain lacking O-antigen biosynthesis genes (SFL1195, S2 Table) and several recombinant serotype Y strains carrying a plasmid with different O-antigen modification genes of *S*. *flexneri* (S2 Table). Plaques were only obtained in control serotype Y strain and in serotype Y strain carrying *O-acetyltransferase* gene (converting serotype Y to 3b, SFL1899), which was consistent with the phage SfMu host range results. This indicates that modification of the O-antigen or deletion of the O-antigen biosynthesis genes results in failure of adsorption of phage SfMu to the host cell, thus suggesting that phage SfMu uses O-antigen as receptor.

**Fig 3:**
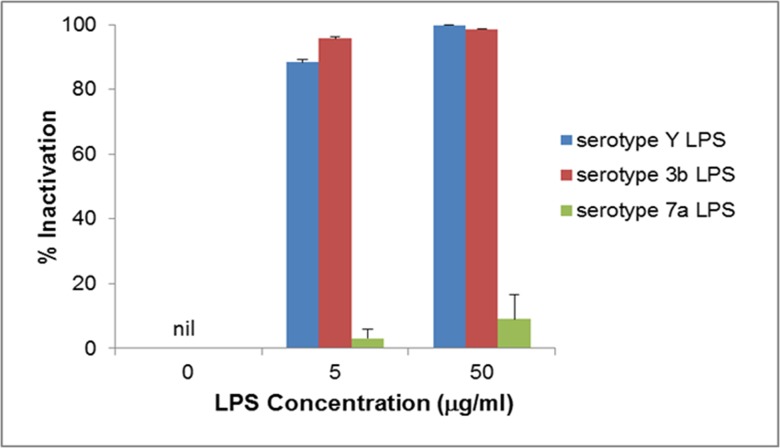
Inactivation of bacteriophage SfMu by LPS derived from *S*. *flexneri* serotypes Y and 3b. Approximately 1x10^3^ PFU of SfMu phage was mixed with varying concentration of the LPS (5 μg or 50 μg) derived from serotype Y, 3b and 7a strains of *S*. *flexneri*. % Inactivation was determined after 1hr of incubation at 37°C. The inhibition data represents the mean ± standard error of 3 experiments.

Results of the plaque assay were further confirmed by performing phage inactivation assay using LPS purified from *S*. *flexneri* serotype Y and 3b strains. As shown in Fig 3, number of plaques significantly reduced in the presence of both Y and 3b LPS, indicating interaction between the LPS and phage SfMu. Moreover, almost complete inactivation of phage was achieved at a concentration of 50 μg with the LPS from either serotype 3b or Y. As the LPS from different serotypes of *S*. *flexneri* vary only in the O-antigen component, LPS of serotype 7a strain was used as a negative control to corroborate that the O-antigen alone and not the core oligosaccharide or both, served as the receptor. As shown in the Fig 3, serotype 7a LPS showed very little/no inactivation in comparison to Y or 3b LPS, thus indicating that the O-antigen of the LPS is the receptor for phage SfMu.

### Prevalence of SfMu phage in *S*. *flexneri*

Since *S*. *flexneri* is not a recognised host for transposable phages, we investigated the prevalence of bacteriophage SfMu in various serotypes of *S*. *flexneri*. A total of 194 wild type *S*. *flexneri* strains tested, comprising isolates from various geographical regions, and represented 15 serotypes of *S*. *flexneri*. All the strains were first screened by performing multiplex PCR using 3 pairs of primers. The primer sets were chosen to target 3 different regions of phage SfMu: *c* repressor-*ner* region (900 bp), middle operon regulator-lysozyme (1.2 kb) and *gin-mom* region (1.5 kb). As shown in S3 Table, all three products of expected sizes were only obtained for the phage SfMu host strain (positive control). However, only the 1.2 kb fragment was obtained in 38 other strains belonging to serotypes 1a, 3a, 3b, 6, Y, Yv, Xv and X. Sequencing of the 1.2 kb PCR product from five these strains revealed that they contained the expected sequence, thus indicating the presence of cryptic SfMu phage in their genome.

The results of multiplex PCR were further validated by performing Southern hybridization. *Eco*RV digested genomic DNA of 34 out of 38 PCR positive strains were probed with DIG-labelled *Eco*RV-digested SfMu DNA. As expected, most of the phage fragments were present in the phage SfMu host (positive control) indicating presence of the phage in the host chromosome (S1 Fig). Additionally, the hybridization pattern of 6 other strains (lanes marked with red asterisk, S1 Fig), belonging to serotype X or Y (originating from Vietnam), was very similar to that of SfMu host, thus suggesting that a complete copy of the SfMu prophage might also be present in these strains. However, the absence of the 900 bp and 1.5 kb PCR products (in multiplex PCR) in these strains suggests that the prophage in their genomes might be related to phage SfMu, but is not identical.

Two bands (4.5 kb and 3.7 kb) appeared to be common in most of the strains, indicating that these strains might contain a remnant of the SfMu prophage in their genomes (S1 Fig). However, except in the SfMu lysogen or the 6 strains containing complete SfMu-like prophage, the 3.7 kb band appears to be a little smaller in size (approx. 3.5 kb), which could be because of restriction site polymorphism.

Taken together, these results indicate that Mu-like phages are not uncommon in *S*. *flexneri*. Moreover, identification of SfMu like prophage in different serotypes of *S*. *flexneri* also suggests that this phage might have a role in transferring diverse sets of virulence genes and other genetic traits from one serotype to another.

## Conclusions

This is the first report on the isolation of a transposable phage, SfMu, from *S*. *flexneri*. The complete genome of phage SfMu was sequenced, characterised, and compared with bacteriophage Mu and other Mu-like phages. Results of comparative analysis revealed that SfMu is the first Mu-like phage isolated from a different genus yet closely related to phage Mu. Further analysis of phage SfMu cell wall receptor revealed that, bacteriophage SfMu recognises LPS O-antigen as its primary receptor for adsorption. This study also identifies presence of complete or cryptic SfMu prophage in various serotypes of *S*. *flexneri*. Further studies on Mu-like phages of *S*. *flexneri* will be useful in understanding the role of these phages in evolution, cellular lifestyle, and pathogenicity of *S*. *flexneri*.

## Supporting Information

S1 Fig Screening of SfMu prophage in various serotypes of *S*. *flexneri* by Southern hybridization. Chromosomal DNA of 34 *S*. *flexneri* strains were digested with *Eco*RV, run on a 0.7% agarose gel, transferred to nylon membrane and probed with DIG-labelled *Eco*RV digested SfMu phage DNA. Agarose gels are shown on the left and their corresponding Southern blots on the right. P represents phage SfMu DNA. L represents SfMu lysogen (positive control). Lanes 1, 3, 4, 12, 18–24, 28–30: serotype 3a strains, lanes 2 and 5: serotype 3b strains, lanes 6, 13, 17, 25, 26, 31: serotype Y strains, lanes7-8: serotype Yv strains, lanes 14–16, 32–34: serotype X strains and lanes 9–11, 27: serotype Xv strains. Numbers in the left margin of agarose gel represent the sizes of the marker (*Eco*RI-digested SPP-1 phage DNA). Red asterisks identify strains containing complete SfMu prophage.
(TIF)

S2 Fig DNA level comparison of bacteriophage SfMu with other Mu-like phages. Whole genome of phage SfMu was compared with that of bacteriophage Mu and 6 other Mu-like phages using progressive Mauve alignment. Coloured outlined blocks surround regions of the genome that aligned to part of another genome. Inside each box, the height of the coloured bars indicates the nucleotide sequence similarity while the regions with no similarity are in white.
(TIF)

S3 Fig Alignment of *E*. *coli* phage Mu tail fiber protein (S’) with SfMu phage tail fiber protein (ORF49). ORF49, tail fiber protein of bacteriophage SfMu was aligned with its homologue in bacteriophage Mu (S’), using Clustal W. Amino acid substitutions are highlighted in pink.
(DOCX)

S1 Table Analysis of predicted *orfs* and proteins of SfMu. (DOCX)

S2 Table *S.flexneri* strains used in the cell wall receptor analysis of phage SfMu. (DOCX)

S3 Table Multiplex PCR for screening SfMu prophage in different serotypes of *S*. *flexneri* isolates from various geographical regions. (DOCX)

## References

[1] Craig NL. Unity in transposition reactions. Science 1995;270: 253–254. 7569973 10.1126/science.270.5234.253

[2] Mizuuchi K. Transpositional recombination: mechanistic insights from studies of mu and other elements. Annu Rev Biochem. 1992;61: 1011–1051. 1323232 10.1146/annurev.bi.61.070192.005051

[3] Faelen M, Toussaint A. Bacteriophage Mu-1: a tool to transpose and to localize bacterial genes. J Mol Biol. 1976;104: 525–539. 781292 10.1016/0022-2836(76)90118-2

[4] Wang X, Higgins NP. 'Muprints' of the lac operon demonstrate physiological control over the randomness of in vivo transposition. Mol Microbiol. 1994;12: 665–677. 7934890 10.1111/j.1365-2958.1994.tb01054.x

[5] Bukhari AI, Froshauer S, Botchan M. Ends of bacteriophage mu DNA. Nature 1976;264: 580–583. 1004602 10.1038/264580a0

[6] Akhverdian VZ, Khrenova EA, Bogush VG, Gerasimova TV, Kirsanov NB. Wide distribution of transposable phages in natural Pseudomonas aeruginosa populations. Genetika 1984;20: 1612–1619. 6094307

[7] Gill GS, Hull RC, Curtiss R 3rd. Mutator bacteriophage D108 and its DNA: an electron microscopic characterization. J Virol. 1981;37: 420–430. 6452532 10.1128/jvi.37.1.420-430.1981 PMC171019

[8] Goudie AD, Lynch KH, Seed KD, Stothard P, Shrivastava S, Wishart DS, et al Genomic sequence and activity of KS10, a transposable phage of the Burkholderia cepacia complex. BMC Genomics 2008;9: 615 10.1186/1471-2164-9-615 19094239 PMC2628397

[9] Summer EJ, Gonzalez CF, Carlisle T, Mebane LM, Cass AM, Saava CG, et al Burkholderia cenocepacia phage BcepMu and a family of Mu-like phages encoding potential pathogenesis factors. J Mol Biol. 2004;340: 49–65. 15184022 10.1016/j.jmb.2004.04.053

[10] Fogg PC, Hynes AP, Digby E, Lang AS, Beatty JT. Characterization of a newly discovered Mu-like bacteriophage, RcapMu, in Rhodobacter capsulatus strain SB1003. Virology 2011;421: 211–221. 10.1016/j.virol.2011.09.028 22018635

[11] Zehr ES, Tabatabai LB, Bayles DO. Genomic and proteomic characterization of SuMu, a Mu-like bacteriophage infecting Haemophilus parasuis. BMC Genomics 2012;13: 331 22823751 10.1186/1471-2164-13-331 PMC3447690

[12] Morgan GJ, Hatfull GF, Casjens S, Hendrix RW. Bacteriophage Mu genome sequence: analysis and comparison with Mu-like prophages in Haemophilus, Neisseria and Deinococcus. J Mol Biol. 2002;317: 337–359. 11922669 10.1006/jmbi.2002.5437

[13] Heidelberg JF, Paulsen IT, Nelson KE, Gaidos EJ, Nelson WC, Read TD, et al Genome sequence of the dissimilatory metal ion-reducing bacterium Shewanella oneidensis. Nat Biotechnol. 2002;20: 1118–1123. 12368813 10.1038/nbt749

[14] Hayashi T, Makino K, Ohnishi M, Kurokawa K, Ishii K, Yokoyama K, et al Complete genome sequence of enterohemorrhagic Escherichia coli O157:H7 and genomic comparison with a laboratory strain K-12. DNA Res. 2001;8: 11–22. 11258796 10.1093/dnares/8.1.11

[15] Kotloff KL, Winickoff JP, Ivanoff B, Clemens JD, Swerdlow DL, Sansonetti PJ, et al Global burden of Shigella infections: implications for vaccine development and implementation of control strategies. Bull World Health Organ. 1999;77: 651–666. 10516787 PMC2557719

[16] Sun Q, Lan R, Wang J, Xia S, Wang Y, Jin D, et al Identification and characterization of a novel Shigella flexneri serotype Yv in China. PLoS One 2013;8: e70238 10.1371/journal.pone.0070238 23936172 PMC3728103

[17] Allison GE, Angeles D, Tran-Dinh N, Verma NK. Complete genomic sequence of SfV, a serotype-converting temperate bacteriophage of Shigella flexneri. J Bacteriol. 2002;184: 1974–1987. 11889106 10.1128/JB.184.7.1974-1987.2002 PMC134923

[18] Casjens S, Winn-Stapley DA, Gilcrease EB, Morona R, Kuhlewein C, Chua JE, et al The chromosome of Shigella flexneri bacteriophage Sf6: complete nucleotide sequence, genetic mosaicism, and DNA packaging. J Mol Biol. 2004;339: 379–394. 15136040 10.1016/j.jmb.2004.03.068

[19] Jakhetia R, Marri A, Stahle J, Widmalm G, Verma NK. Serotype-conversion in Shigella flexneri: identification of a novel bacteriophage, Sf101, from a serotype 7a strain. BMC Genomics 2014;15: 742 10.1186/1471-2164-15-742 25174528 PMC4159516

[20] Jakhetia R, Talukder KA, Verma NK. Isolation, characterization and comparative genomics of bacteriophage SfIV: a novel serotype converting phage from Shigella flexneri. BMC Genomics 2013;14: 677 10.1186/1471-2164-14-677 24090466 PMC3851460

[21] Mavris M, Manning PA, Morona R. Mechanism of bacteriophage SfII-mediated serotype conversion in Shigella flexneri. Mol Microbiol. 1997;26: 939–950. 9426131 10.1046/j.1365-2958.1997.6301997.x

[22] Sun Q, Lan R, Wang Y, Wang J, Wang Y, Li P, et al Isolation and genomic characterization of SfI, a serotype-converting bacteriophage of Shigella flexneri. BMC Microbiol. 2013;13: 39 10.1186/1471-2180-13-39 23414301 PMC3636060

[23] Adams MM, Allison GE, Verma NK. Type IV O antigen modification genes in the genome of Shigella flexneri NCTC 8296. Microbiology 2001;147: 851–860. 11283281 10.1099/00221287-147-4-851

[24] Lindberg AA, Karnell A, Stocker BA, Katakura S, Sweiha H, Reinholt FP, et al Development of an auxotrophic oral live Shigella flexneri vaccine. Vaccine 1988;6: 146–150. 2838986 10.1016/s0264-410x(88)80018-5

[25] Sambrook J, Fritsch E F, Maniatis T. Molecular Cloning: a Laboratory Manual, 2nd edn. Cold Spring Harbor, NY: Cold Spring Harbor Laboratory; 1989.

[26] Santos SB, Carvalho CM, Sillankorva S, Nicolau A, Ferreira EC, Azeredo J, et al The use of antibiotics to improve phage detection and enumeration by the double-layer agar technique. BMC Microbiol. 2009;9: 148 10.1186/1471-2180-9-148 19627589 PMC2728735

[27] Westphal O JK. Bacterial lipopolysaccharides. Extraction with phenol-water and further applications of the procedure. Methods Carbohydr Chem. 1965;5: 83–90.

[28] Sandulache R, Prehm P, Kamp D. Cell wall receptor for bacteriophage Mu G(+). J Bacteriol. 1984;160: 299–303. 6384194 10.1128/jb.160.1.299-303.1984 PMC214716

[29] Zerbino DR, Birney E. Velvet: algorithms for de novo short read assembly using de Bruijn graphs. Genome Res. 2008;18: 821–829. 10.1101/gr.074492.107 18349386 PMC2336801

[30] Schattner P, Brooks AN, Lowe TM. The tRNAscan-SE, snoscan and snoGPS web servers for the detection of tRNAs and snoRNAs. Nucleic Acids Res. 2005;33: W686–689. 15980563 10.1093/nar/gki366 PMC1160127

[31] Gautheret D, Lambert A. Direct RNA motif definition and identification from multiple sequence alignments using secondary structure profiles. J Mol Biol. 2001;313: 1003–1011. 11700055 10.1006/jmbi.2001.5102

[32] Darling AE, Mau B, Perna NT. ProgressiveMauve: multiple genome alignment with gene gain, loss and rearrangement. PLoS One 2010;5: e11147 10.1371/journal.pone.0011147 20593022 PMC2892488

[33] Thompson JD, Higgins DG, Gibson TJ. CLUSTAL W: improving the sensitivity of progressive multiple sequence alignment through sequence weighting, position-specific gap penalties and weight matrix choice. Nucleic Acids Res. 1994;22: 4673–4680. 7984417 10.1093/nar/22.22.4673 PMC308517

[34] Bradley DE. Ultrastructure of bacteriophage and bacteriocins. Bacteriol Rev. 1967;31: 230–314. 4865539 10.1128/br.31.4.230-314.1967 PMC408286

[35] Vogel JL, Li ZJ, Howe MM, Toussaint A, Higgins NP. Temperature-sensitive mutations in the bacteriophage Mu c repressor locate a 63-amino-acid DNA-binding domain. J Bacteriol. 1991;173: 6568–6577. 1833382 10.1128/jb.173.20.6568-6577.1991 PMC208994

[36] Symonds N, Toussaint A, Van de Putte P, Howe MM. Phage Mu. Cold Spring Harbor, N.Y.: Cold Spring Harbor Press; 1987.

[37] Giphart-Gassler M, Wijffelman C, Reeve J. Structural polypeptides and products of late genes of bacteriophage Mu: characterization and functional aspects. J Mol Biol. 1981;145: 139–163. 6455529 10.1016/0022-2836(81)90338-7

[38] Grundy FJ, Howe MM. Morphogenetic structures present in lysates of amber mutants of bacteriophage Mu. Virology 1985;143: 485–504. 3904174 10.1016/0042-6822(85)90388-5

[39] Kamp D, Kahmann R. The relationship of two invertible segments in bacteriophage Mu and Salmonella typhimurium DNA. Mol Gen Genet. 1981;184: 564–566. 7038403 10.1007/BF00352543

[40] van de Putte P, Cramer S, Giphart-Gassler M. Invertible DNA determines host specificity of bacteriophage mu. Nature 1980;286: 218–222. 6250048 10.1038/286218a0

[41] Mertens G, Hoffmann A, Blocker H, Frank R, Kahmann R. Gin-mediated site-specific recombination in bacteriophage Mu DNA: overproduction of the protein and inversion in vitro. EMBO J. 1984;3: 2415–2421. 16453561 10.1002/j.1460-2075.1984.tb02148.x PMC557702

[42] Sandulache R, Prehm P, Expert D, Toussaint A, Kamp D. The cell wall receptor for bacteriophage Mu G(−) in Erwinia and Escherichia coli C. FEMS Microbiology Letters 1985;28: 307–310.

[43] Iida S, Hiestand-Nauer R. Localized conversion at the crossover sequences in the site-specific DNA inversion system of bacteriophage P1. Cell 1986;45: 71–79. 3513965 10.1016/0092-8674(86)90539-8

[44] Schmucker R, Ritthaler W, Stern B, Kamp D. DNA inversion in bacteriophage Mu: characterization of the inversion site. J Gen Virol. 1986;67 (Pt 6): 1123–1133. 3011972 10.1099/0022-1317-67-6-1123

[45] Comeau AM, Bertrand C, Letarov A, Tétart F, Krisch HM. Modular architecture of the T4 phage superfamily: A conserved core genome and a plastic periphery. Virology 2007;362: 384–396. 17289101 10.1016/j.virol.2006.12.031

[46] Montag D, Riede I, Eschbach ML, Degen M, Henning U. Receptor-recognizing proteins of T-even type bacteriophages. Constant and hypervariable regions and an unusual case of evolution. J Mol Biol. 1987;196: 165–174. 2958637 10.1016/0022-2836(87)90519-5

[47] Dupont K, Vogensen FK, Neve H, Bresciani J, Josephsen J. Identification of the receptor-binding protein in 936-species lactococcal bacteriophages. Appl Environ Microbiol. 2004;70: 5818–5824. 15466519 10.1128/AEM.70.10.5818-5824.2004 PMC522089

[48] Le S, He X, Tan Y, Huang G, Zhang L, Lux R, et al Mapping the tail fiber as the receptor binding protein responsible for differential host specificity of Pseudomonas aeruginosa bacteriophages PaP1 and JG004. PLoS One 2013;8: e68562 10.1371/journal.pone.0068562 23874674 PMC3706319

[49] Wang J, Hofnung M, Charbit A. The C-terminal portion of the tail fiber protein of bacteriophage lambda is responsible for binding to LamB, its receptor at the surface of Escherichia coli K-12. J Bacteriol. 2008;182: 508–512. 10.1128/jb.182.2.508-512.2000 PMC94303 10629200

