# Supplementary figures and images for: Identification and Molecular Characterisation of a Novel Mu-Like Bacteriophage, SfMu, of Shigella flexneri
Source: PLoS One. 2015 Apr 22;10(4):e0124053. doi: 10.1371/journal.pone.0124053 (PMC4406740; doi:10.1371/journal.pone.0124053)

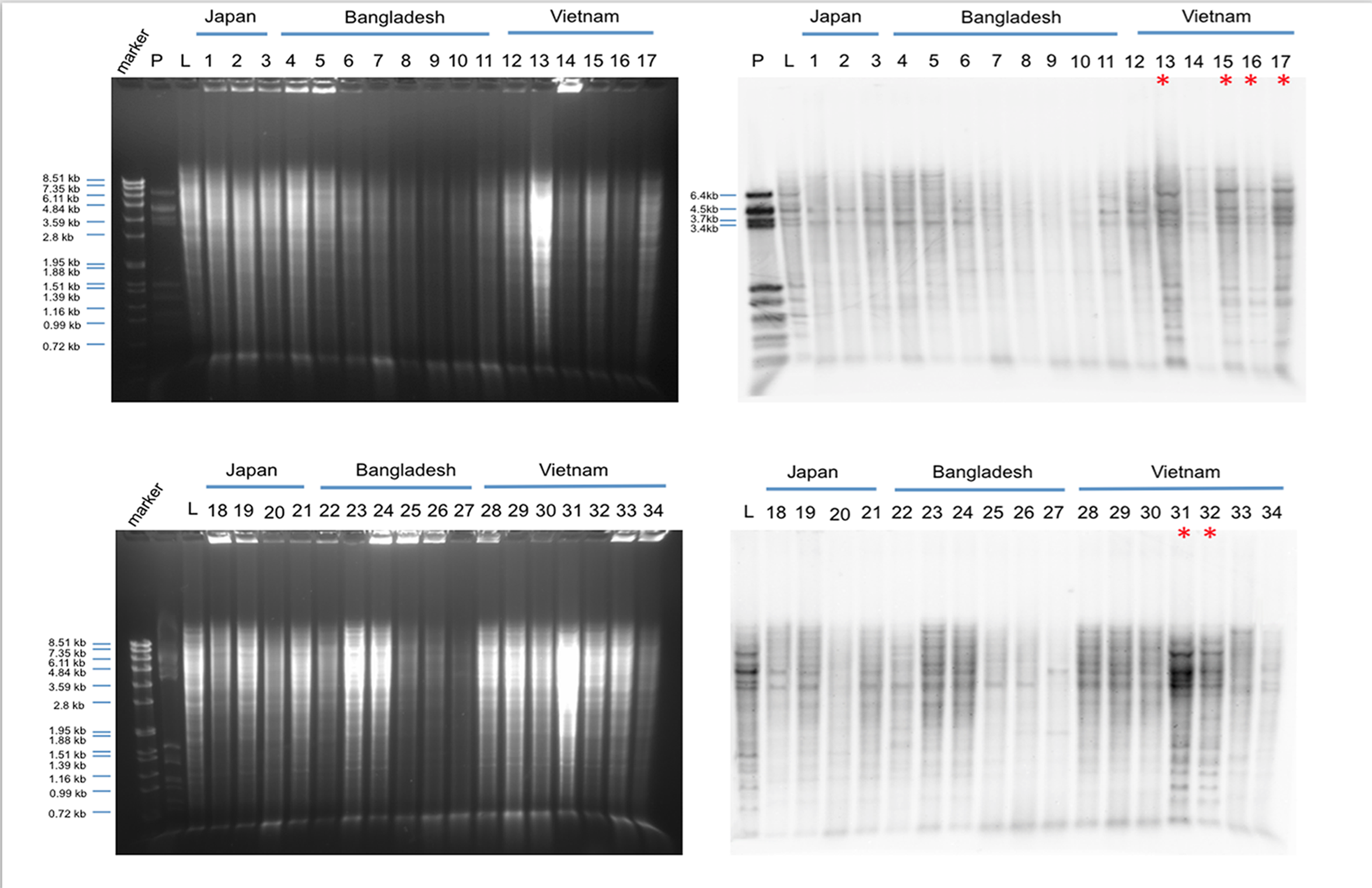

Supplement: S1 Fig — Chromosomal DNA of 34 S. flexneri strains were digested with EcoRV, run on a 0.7% agarose gel, transferred to nylon membrane and probed with DIG-labelled EcoRV digested SfMu phage DNA. Agarose gels are shown on the left and their corresponding Southern blots on the right. P represents phage SfMu DNA. L represents SfMu lysogen (positive control). Lanes 1, 3, 4, 12, 18–24, 28–30: serotype 3a strains, lanes 2 and 5: serotype 3b strains, lanes 6, 13, 17, 25, 26, 31: serotype Y strains, lanes7-8: serotype Yv strains, lanes 14–16, 32–34: serotype X strains and lanes 9–11, 27: serotype Xv strains. Numbers in the left margin of agarose gel represent the sizes of the marker (EcoRI-digested SPP-1 phage DNA). Red asterisks identify strains containing complete SfMu prophage. (TIF) [file pone.0124053.s001.tif]

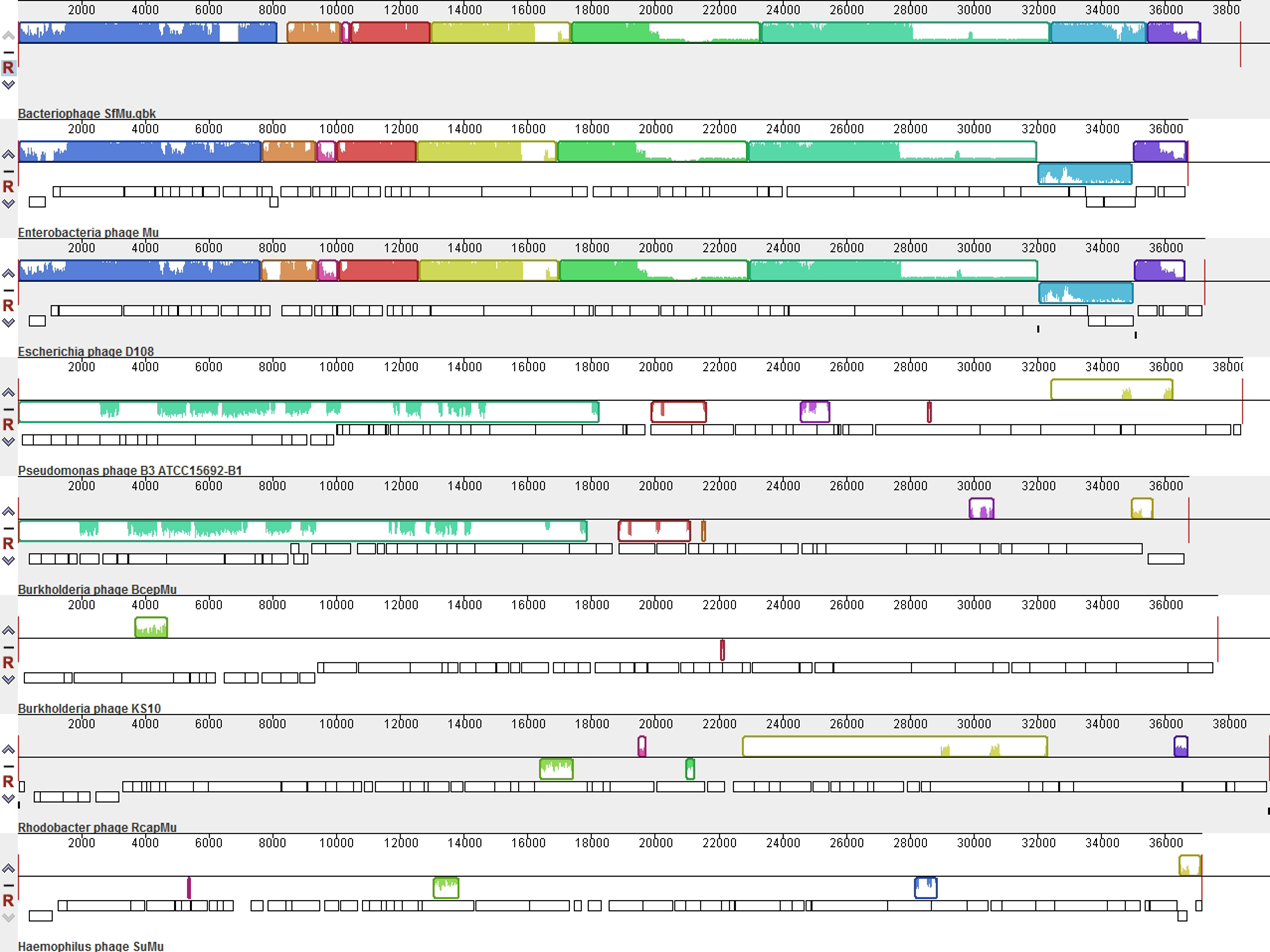

Supplement: S2 Fig — Whole genome of phage SfMu was compared with that of bacteriophage Mu and 6 other Mu-like phages using progressive Mauve alignment. Coloured outlined blocks surround regions of the genome that aligned to part of another genome. Inside each box, the height of the coloured bars indicates the nucleotide sequence similarity while the regions with no similarity are in white. (TIF) [file pone.0124053.s002.tif]
