# Supplementary material for: Identification and Molecular Characterisation of a Novel Mu-Like Bacteriophage, SfMu, of Shigella flexneri
Source: PLoS One. 2015 Apr 22;10(4):e0124053. doi: 10.1371/journal.pone.0124053 (PMC4406740; doi:10.1371/journal.pone.0124053)
Supplement: S3 Fig — ORF49, tail fiber protein of bacteriophage SfMu was aligned with its homologue in bacteriophage Mu (S’), using Clustal W. Amino acid substitutions are highlighted in pink. (DOCX) [file pone.0124053.s003.docx]

Mu MFYIDNDSGVTVMPPVSAQRSAIVRWFSEGDGNNVITWPGMDWFNIVQAELLNTLEEAGI 60

SfMu MFYIDNDSGVTVMPPVSAQRSAIVRWFSEGDGNNVITWPGMDWFNIVQAELLNTLEEAGI 60

Mu QPDKTKLNQLALSIKAIMSNNALLIKNNLSEIKTAGASAQRTARENLDIYDASLNKKGLV 120

SfMu QPDKTKLNQLALSIKAIMSNNALLIKNNLSEIKTAGASAQRTARENLDIYDASLNKKGLV 120

Mu QLTSATDSPSETLAATAKAVKIAMDNANARLAKDRNGADIPNKPLFIQNLGLQETVNQAS 180

SfMu QLTSATDSPSETLAATAKAVKIAMDNASARLAKDRNGADIPNKPLFIQNVGLQETVNQAS 180

Mu GALQQNQNGADIPGKDTFTKNIGACRAYSAWLNIGGDSQVWTTAQFISWLESQGAFNHPY 240

SfMu GALQKNQNGADIPGKDTFTKNIGACRAYSAWVDIGGDSQVWTTAQFISWLESQGAFNHPY 240

Mu WMCKGSWAYANNKVITDTGCGNICLAGAVVEVIGTRGAMTIRVTTPSTSSGGGITNAQFT 300

SfMu WMCKGSWAYANNKVITDTGCGNICLAGAVVEVIGTRGAMTIRVTTPSTSSGGGITNAQFT 300

Mu YINHGDAYAPGWRRDYNTKNQQPAFALGQTGSTVGNDKAVGWNWNSGVYNANIGGASTLI 360

SfMu YINHGNAYAPGWRRDYNTKNQQPAFALGQTGNTVGNDKAVGWNWNSGVYDADISGASTLI 360

Mu LHFNMNTGSCPAVQFRVNYRNGGIFYRSARDGYGFEADWSEIYTTTRKPSAGDVGAYTQA 420

SfMu LHFNKNTGSCPAVQFRVNYKNGGIFYRSARDGYGFEAGWSEFYTTTRKPSARDVGAYTQA 420

Mu ECNSRFITGIRLGGLSSVQTWNGPGWSDRSGYVVTGSVNGNRDELIDTTQARPIQYCING 480

SfMu ECNSRFITGIRLGGLSSVRTWNGPGWSDRSGYVVTGSVNSNRDELIDTTQARPVQYCING 480

Mu TWYNAGSI 488

SfMu TWYNAGSI 488
