# Supplementary material for: Identification and Molecular Characterisation of a Novel Mu-Like Bacteriophage, SfMu, of Shigella flexneri
Source: PLoS One. 2015 Apr 22;10(4):e0124053. doi: 10.1371/journal.pone.0124053 (PMC4406740; doi:10.1371/journal.pone.0124053)
Supplement: S1 Table — (DOCX) [file pone.0124053.s004.docx]

S1 Table: Analysis of predicted *orfs* and proteins of SfMu.

| ***orf*** | **Product** | **Region** | **Size (a.a)** | **Related products (sizes)** | **Accession no.** | **BlastP evalue (% identity)** |
| --- | --- | --- | --- | --- | --- | --- |
| 1 | c-repressor protein | complement(326..850) | 174 | mu DNA-binding domain protein [174aa, Shigella boydii 5216-82], repressor protein [174aa, Escherichia coli E24377A] | ZP_12339296.1, YP_001462168.1 | 1E-124 (100), 1e-110 (93) |
| 2 | DNA binding protein Ner | 1040..1261 | 73 | DNA-binding protein Ner [73aa, Shigella boydii 5216-82], Ner [73aa, Escherichia phage D108] | ZP_12339295.1, YP_003335750.1 | 6e-46 (100), 2e-45 (98) |
| 3 | Transposase | 1269..3257 | 662 | transposase [662aa, Shigella boydii 5216-82], DNA transposition protein A [662aa, Escherichia phage D108] | ZP_12339294.1, YP_001462167.1 | 0.0 (100), 0.0(98) |
| 4 | DNA transposition protein | 3296..4234 | 312 | DNA transposition protein [312aa, Enterobacteria phage Mu], DNA transposition protein [312aa, Shigella boydii 5216-82] | NP_050608.1 | 0.0 (98) |
| 5 | Unknown | 4250..4477 | 75 | protein cim [75aa, Shigella boydii 5216-82], Kil [Escherichia phage D108] | ZP_12341830.1, ZP_03062883.1 | 7E-48 (100), 3e-47 (100) |
| 6 | Unknown | 4480..4710 | 76 | conserved hypothetical protein [76aa, Escherichia coli B171], hypothetical protein SB521682_4960 [76aa, Shigella boydii 5216-82] | ZP_03062875.1, ZP_03003525.1 | 5e-47 (100), 3e-46 (99) |
| 7 | Unknown | 4722..4985 | 87 | hypothetical protein SB521682_5313 [87aa, Shigella boydii 5216-82], hypothetical protein Mup07 [87aa, Enterobacteria phage Mu] | ZP_12336979.1, NP_050611.1 | 6E-52 (98), 7e-51 (97) |
| 8 | Unknown | 5000..5419 | 139 | hypothetical protein SB521682_5312 [147aa, Shigella boydii 5216-82], hypothetical protein SFK272_3569 [139aa, Shigella flexneri K-272] | ZP_12336982.1, ZP_12362783.1 | 1E-97 (100), 2e-96(99) |
| 9 | Unknown | 5432..5719 | 95 | hypothetical protein SB521682_5311 [95, Shigella boydii 5216-82], hypothetical protein SFK272_3568 [95aa, Shigella flexneri K-272] | ZP_12336980.1, ZP_12362782.1 | 9e-63 (100), 5e-62 (100) |
| 10 | Host nuclease inhibitor protein | 5738..6262 | 174 | host-nuclease inhibitor protein gam [174aa, Shigella boydii 5216-82], bacteriophage Mu Gam like protein [174aa, Escherichia coli 53638] | ZP_12336978.1, ZP_02999360.1 | 5E-121 (100), 8e-119 (99) |
| 11 | Unknown | 6354..6908 | 184 | hypothetical protein SFJ1713_3220 [184aa, Shigella flexneri J1713],hypothetical protein SB521682_5309 [182aa, Shigella boydii 5216-82] | ZP_12271468.1, ZP_12336976.1 | 3e-135 (100), 4e-133 (100) |
| 12 | Unknown | 6912..7442 | 176 | hypothetical protein SB521682_5308 [176aa, Shigella boydii 5216-82], hypothetical protein [176aa, Escherichia phage D108] | ZP_12336975.1 , YP_003335759.1 | 4e-126 (100), 8e-113 (99) |
| 13 | Unknown | 7957..8139 | 60 | hypothetical protein [60aa, Escherichia phage D108], hypothetical protein SB521682_5330 [60aa, Shigella boydii 5216-82] | ZP_03062600.1 | 5E-36 (100) |
| 14 | Unknown | 8141..8449 | 102 | hypothetical protein SB521682_5329 [102aa, Shigella boydii 5216-82], conserved hypothetical protein [102aa, Escherichia coli B171] | ZP_12337000.1, ZP_03062584.1 | 3e-67 (100), 5e-67 (100) |
| 15 | Unknown | 8446..8748 | 100 | e15 [100aa, Escherichia coli B171], hypothetical protein SB521682_5328 [100aa, Shigella boydii 5216-82] | ZP_03062580.1 | 6e-65 (100) |
| 16 | Unknown | complement(8687..8953) | 88 | hypothetical protein Mup15 [88aa, Enterobacteria phage Mu], hypothetical protein [88aa, Escherichia coli KO11FL] | NP_050619.1 | 8E-54 (98) |
| 17 | Unknown | 9023..9574 | 183 | hypothetical protein ECDEC13C_2795 [183aa, Escherichia coli DEC13C], hypothetical protein ECDEC14D_1520 [183aa, Escherichia coli DEC14D] | ZP_13897019.1, ZP_13926601.1 | 3E-133 (100), 1e-132(99) |
| 18 | Middle operon regulator | 9571..9960 | 129 | Mor [129aa, Escherichia phage D108], middle operon regulator [129aa, Shigella boydii 5216-82] | YP_003335764.1 | 1e-88(100) |
| 19 | Unknown | 10038..10268 | 76 | conserved hypothetical protein [76aa, Escherichia coli 53638], hypothetical protein Mup18 [72aa, Enterobacteria phage Mu] | ZP_02998965.1, NP_050622.1 | 1E-49 (100), 3e-42 (95) |
| 20 | Unknown | 10249..10368 | 39 | conserved hypothetical protein [39aa, Escherichia coli B171], hypothetical protein Mup20 [39aa, Enterobacteria phage Mu] | ZP_03062609.1, NP_050624.1 | 2e-17 (100), 2e-16 (97) |
| 21 | Regulator of late transcription | 10381..10803 | 140 | mor transcription activator family protein [140aa, Escherichia coli DEC1D], late gene transcriptional activator [140aa, Escherichia phage D108] | ZP_13562710.1, ZP_03062589.1 | 5e-98 (100), 5e-97(199) |
| 22 | Lysozyme | 10898..11413 | 171 | lysozyme [171aa, Escherichia coli DEC13D], Lys [171aa, Escherichia phage D108] | YP_003335769.1 | 4e-121 (98) |
| 23 | Putative Rz | 11397..11783 | 128 | conserved hypothetical protein [128aa, Escherichia coli B171], hypothetical protein ECDEC1D_4247 [128aa, Escherichia coli DEC1D] | ZP_03062601.1 | 3E-86 (99) |
| 24 | Unknown | 11942..12136 | 64 | hypothetical protein SB521682_5320 [64aa, Shigella boydii 5216-82], hypothetical protein Mup24 [64aa, Enterobacteria phage Mu] | ZP_12336996.1, NP_050628.1 | 3E-39 (100), 1e-38 (100) |
| 25 | Unknown | 12136..12438 | 100 | hypothetical protein ECDEC13E_2624 [97aa, Escherichia coli DEC13E], hypothetical protein Mup25 [99aa, Enterobacteria phage Mu] | ZP_13907083.1, NP_050629.1 | 9E-59 (95), 3e-58 (95) |
| 26 | Unknown | 12435..12725 | 96 | hypothetical protein Mup26 [96aa, Enterobacteria phage Mu], hypothetical protein [96aa, Escherichia phage D108] | NP_050630.1 | 1E-62 (100) |
| 27 | Unknown | 12737..13312 | 191 | hypothetical protein Mup27 [Enterobacteria phage Mu] , conserved hypothetical protein [Escherichia phage D108] | NP_050631.1 | 8E-133 (100) |
| 28 | Unknown | 13320..14975 | 551 | portal protein [551aa, Escherichia coli DEC12B],hypothetical protein [551aa, Escherichia phage D108] | ZP_03062588.1, YP_003335775.1 | 0.0 (99), 0.0 (100) |
| 29 | Portal protein | 14975..16513 | 512 | portal protein [512aa, Escherichia phage D108], hypothetical protein A1SM_03318 [512aa, Escherichia coli KTE57] | YP_003335776.1, ZP_19820494.1 | 0.0 (100), 0.0 (99) |
| 30 | Phage head morphogenesis | 16494..17813 | 439 | F protein (gpF) (Protein gp30) [439aa, Escherichia coli M605], phage head morphogenesis, SPP1 gp7 family domain protein [439aa, Escherichia coli DEC12D] | ZP_08351596.1, ZP_03062582.1 | 0.0 (98), 0.0 (98) |
| 31 | Virion morphogenesis protein | 17810..18280 | 156 | putative virion morphogenesis protein [156aa, Enterobacteria phage Mu], virion morphogenesis protein [156aa, Escherichia phage D108] | NP_050635.1, ZP_03000818.1 | 5E-108 (99), 1e-106 (98) |
| 32 | Protease | 18477..19562 | 361 | protease [361aa, Escherichia phage D108], protein gp32 [361aa, Escherichia coli M605] | YP_003335780.1, ZP_08351598.1 | 0.0 (99), 0.0(99) |
| 33 | Scaffold protein | 19011..19562 | 183 | scaffold protein [183aa, Escherichia phage D108], scaffold protein [183aa, Enterobacteria phage Mu] | YP_003335781.1, NP_050637.1 | 3E-120 (100), 6e-109 (98) |
| 34 | Major head subunit | 19559..20476 | 305 | major head subunit [305aa, Escherichia coli 53638], Phage major capsid protein [Salmonella enterica subsp. enterica serovar Adelaide str. A4-669] | ZP_03002840.1 | 0.0 (100) |
| 35 | Unknown | 20543..20953 | 136 | protein gp35 [136aa, Escherichia coli DEC1D], hypothetical protein ECSE_2583 [136aa, Escherichia coli SE11] | ZP_13562697.1, YP_002293858.1 | 2E-84 (95), 1e-83 (96) |
| 36 | Unknown | 20950..21372 | 140 | hypothetical protein SB521682_5346 [Shigella boydii 5216-82], hypothetical protein ECSE_2582 [Escherichia coli SE11] | ZP_12337017.1, YP_002293857.1 | 5E-98 (100), 1e-76 (87) |
| 37 | Unknown | 21372..21920 | 182 | hypothetical protein SB521682_5345 [182aa, Shigella boydii 5216-82], hypothetical protein ECSE_2581 [182aa, Escherichia coli SE11] | ZP_12337015.1, YP_002293856.1 | 2E-132 (100), 7e-127 (97) |
| 38 | Unknown | 21907..22110 | 67 | hypothetical protein SB521682_5344 [67aa, Shigella boydii 5216-82], hypothetical protein Mup38 [67aa, Enterobacteria phage Mu] | ZP_03002787.1, NP_050642.1 | 1e-39 (100), 8e-39 (98) |
| 39 | Tail sheath protein | 22107..23594 | 495 | bacteriophage Mu tail sheath protein family protein [495aa, Shigella boydii 5216-82], tail sheath protein [Escherichia phage D108] | ZP_12337009.1, YP_003335787.1 | 0.0 (100), 0.0 (98) |
| 40 | Tail tube protein | 23604..23960 | 118 | tail tube protein [118aa, Shigella boydii 5216-82],hypothetical protein Mup40 [Enterobacteria phage Mu] | ZP_03001333.1, NP_050644.1 | 5E-80 (100), 5e-75 (96) |
| 41 | Unknown | 23970..24404 | 144 | putative phage protein [144aa, Escherichia coli 53638], protein gp41 [144aa, Shigella boydii 5216-82] | ZP_02999171.1 | 3E-96 (100) |
| 42 | Tail tape measure protein | 24549..26621 | 690 | protein gp42 [690aa, Escherichia coli 53638], tape measure domain protein [691aa, Escherichia coli DEC13C] | ZP_02999836.1, ZP_13896993.1 | 0.0 (99), 0.0 (96) |
| 43 | DNA circulation protein | 26626..28113 | 495 | DNA circulation family protein [495aa, Shigella boydii 5216-82], DNA circulation protein (64 kDa virion protein) [495aa, Escherichia coli M605] | ZP_12337005.1, ZP_08351608.1 | 0.0 (99), 0.0 (99) |
| 44 | Tail protein | 28106..29239 | 377 | Prophage tail protein [377aa, Salmonella enterica subsp. enterica serovar Adelaide str. A4-669], bacteriophage Mu P protein [377aa, Escherichia coli M605] | ZP_12112722.1, ZP_08351609.1 | 0.0 (98), 0.0 (98) |
| 45 | Baseplate assembly protein | 29227..29820 | 197 | baseplate assembly protein [Escherichia phage D108], phage baseplate assembly protein V [Escherichia coli KTE227] | YP_003335794.1, ZP_20195044.1 | 2E-142(100), 2e-141 (99) |
| 46 | Unknown | 29817..30254 | 145 | hypothetical protein Mup46 [145aa, Enterobacteria phage Mu], conserved hypothetical protein [145aa, Escherichia phage D108] | NP_050650.1 | 3E-98 (100) |
| 47 | Baseplate J like protein | 30255..31337 | 360 | baseplate J-like protein [360aa, Escherichia coli 53638], hypothetical protein [360aa, Escherichia phage D108] | ZP_03001338.1, YP_003335796.1 | 0.0 (99), 0.0(99) |
| 48 | Unknown | 31328..31870 | 180 | protein gp48 [Escherichia coli LT-68], hypothetical protein [Escherichia phage D108] | ZP_11506119.1, YP_002293845.1 | 4E-131 (100), 1e-130 (99) |
| 49 | Tail fiber protein | 31870..33336 | 488 | Tail fiber protein [488aa, Escherichia coli W], phage tail fiber repeat family protein [486aa, Escherichia coli DEC12D] | YP_006123055.1, ZP_13874761.1 | 0.0 (98), 0.0 (94) |
| 50 | Tail fiber assembly protein | 33339..33872 | 177 | caudovirales tail fibre assembly family protein [177aa, Shigella sonnei 53G], tail fiber assembly protein [177aa, Enterobacteria phage Mu] | YP_005456190.1, NP_602302.1 | 7e-128 (100), 6E-125 (99) |
| 51 | Tail fiber assembly protein | complement(33901..34428) | 175 | tail fiber assembly protein [175aa, Escherichia coli O103:H2 str. CVM9450], caudovirales tail fibre assembly family protein [175aa, Escherichia coli OK1180] | ZP_14390287.1, ZP_11508830.1 | 9E-121 (97), 3e-120 (97) |
| 52 | Tail fiber protein | complement(34430..35395) | 321 | Bcv [558aa, Shigella sonnei], tail fiber protein [328aa, Shigella sonnei 3226-85] | BAA00552.1, ZP_14859274.1 | 0.0 (100), 0.0 (100) |
| 53 | Gin | 35521..36114 | 197 | G region invertase [197aa, Escherichia phage D108], DNA-invertase [197aa, Shigella dysenteriae Sd197] | YP_403513.1 | 3E-141 (100) |
| 54 | Com | 36197..36385 | 62 | Com [62aa, Enterobacteria phage Mu], Com [62aa, Escherichia phage D108] | NP_050656.1 | 4E-37 (100) |
| 55 | Mom | 36306..37031 | 241 | phage DNA modification protein [241aa, Escherichia coli SE11], Mom [241aa, Enterobacteria phage Mu] | YP_002293839.1, NP_050657.1 | 3E-178 (100), 5e-178 (100) |
