# Supplementary material for: Identification and Molecular Characterisation of a Novel Mu-Like Bacteriophage, SfMu, of Shigella flexneri
Source: PLoS One. 2015 Apr 22;10(4):e0124053. doi: 10.1371/journal.pone.0124053 (PMC4406740; doi:10.1371/journal.pone.0124053)
Supplement: S2 Table — (DOCX) [file pone.0124053.s005.docx]

S2 Table: *S. flexneri* strains used in the cell wall receptor analysis of phage SfMu.

| **Strain** | **Characteristics** | **Source** |
| --- | --- | --- |
| SFL124 | Attenuated serotype Y vaccine candidate (∆*aro*D) | [24] |
| SFL1195 | SFL124 ∆*rfb* | I. Falt, Karolinska Institute |
| SFL1244 | SFL124 expressing *S. flexneri* *gtrI* cluster cloned in pUC18 (converted to serotype 1a) | N. Verma, ANU |
| SFL1256 | SFL124 expressing *S. flexneri* *gtrV* cluster cloned in pUC18 (converted serotype Y to 5a) | N. Verma, ANU |
| SFL1264 | SFL124 expressing *S. flexneri* *gtrIV* cluster cloned in pUC18 (converted serotype Y to 4a) | N. Verma, ANU |
| SFL1344 | SFL124 expressing *S. flexneri* *gtrX* cluster cloned in pBS KS (converted serotype Y to X) | N. Verma, ANU |
| SFL1420 | SFL124 expressing *S. flexneri* *gtrII* cluster in cloned in pBC SK (converted serotype Y to 2a) | N. Verma, ANU |
| SFL1899 | SFL124 expressing *S. flexneri* *oac* in pBC SK (converted serotype Y to 3b) | N. Verma, ANU |
