# Supplementary material for: Identification and Molecular Characterisation of a Novel Mu-Like Bacteriophage, SfMu, of Shigella flexneri
Source: PLoS One. 2015 Apr 22;10(4):e0124053. doi: 10.1371/journal.pone.0124053 (PMC4406740; doi:10.1371/journal.pone.0124053)
Supplement: S3 Table — (DOCX) [file pone.0124053.s006.docx]

S3 Table: Multiplex PCR for screening SfMu prophage in different serotypes of *S. flexneri* isolates from various geographical regions.

| **Region** | **Serotype** | **Number of strains** | **Number of strains positive for:** | | |
| --- | --- | --- | --- | --- | --- |
|  |  |  | **C repressor-Ner fragment**  **(0.9 kb)** | **Middle operon regulator -lysozyme fragment**  **(1.2 kb)** | **Gin-mom fragment**  **(1.5 kb)** |
| Bangladesh | 1a  1b  2a  2b  3a  3b  4a  4av  5a  6  7a  X  Xv  Y  Yv | 4  4  5  5  5  2  1  4  1  9  5  1  4  3  2 | -  -  -  -  -  -  -  -  -  -  -  -  -  -  - | -  -  -  -  5  1  -  -  -  1  -  -  4  3  2 | -  -  -  -  -  -  -  -  -  -  -  -  -  -  - |
| Japan | 1a  1b  2a  2b  3a  3b  4a  4av  6  X  Y | 2  9  7  5  7  1  2  2  5  1  4 | -  -  -  -  -  -  -  -  -  -  - | -  -  -  -  7  1  -  -  -  -  - | -  -  -  -  -  -  -  -  -  -  - |
| Vietnam | 1a  2a  3a  3b  4a  4av  6  7a  X  Y | 19  1  4  1  11  7  2  7  7  25 | -  -  -  -  -  -  -  -  -  - | 1  -  4  -  -  -  6  3  -  - | -  -  -  -  -  -  -  -  -  - |
| Sweden | 7a  7b (1d) | 2  2 | -  - | -  - | -  - |
| UK | 1a  7a  4a (NCTC)  4b (NCTC) | 1  3  1  1 | -  -  -  - | -  -  -  - | -  -  -  - |
| SfMu host (SFL2241) | 4a |  | + | + | + |
| Total | | 194 |  | 38 |  |
